# Older person’s experiences of benefits gained from the support and advice given during preventive home visits

**DOI:** 10.1111/scs.12923

**Published:** 2020-10-30

**Authors:** Anna Nivestam, Pia Petersson, Albert Westergren, Maria Haak

**Affiliations:** ^1^ Department of Nursing and Health Sciences Kristianstad University Kristianstad Sweden; ^2^ Department of Health Sciences Lund University Lund Sweden

**Keywords:** community care, dialogue, empowerment, experience, older adults, older people, person centredness, prevention, promotion, qualitative

## Abstract

**Background:**

Life expectancy is increasing all over the world. To be able to support this positive development, health interventions focusing on promotion and prevention are crucial. Preventive home visits represent one example of a health intervention which addresses both promotion through a supportive dialogue and prevention by giving advice. However, to give support and advice that older persons experience as beneficial, there is a need for more research.

**Aim:**

The aim of this study was to explore older person’s experiences of the benefits gained from the support and advice given during the preventive home visit.

**Method:**

Individual semi‐structured interviews were conducted with 13 older persons, median age 77 years old, living at home, who had received a preventive home visit. The interviews were analysed with content analysis.

**Findings:**

The overarching theme *Becoming empowered and recognised as a person* was experienced as the major benefit of the support and advice given during the preventive home visit. The support and advice generated conditions for the person to become empowered, by contributing to a feeling of control and preparedness for the future. Furthermore, the support and advice given contributed to a feeling of becoming recognised as a person, as an outcome of the supportive dialogue and the assessment of their health, behaviour and their surrounding environment.

**Conclusion:**

The support and advice given during the preventive home visit were experienced as person‐centred, and conditions for becoming empowered were created. In order to create a positive outcome from the support and advice given during the preventive home visit, it seems important to focus on providing both a supportive dialogue and a structured assessment.

## Introduction

Life expectancy is increasing all over the world (1), and in Sweden, the population above the age of 80 will more than double in the coming decades (2). This demographic development can be viewed as challenging since older persons have an increased risk of disease (3) and comorbidity (4) which might lead to increased costs for health services. However, older persons can also be viewed as capable (5). They can be viewed as a resource, and as persons who can manage challenges and keep on contributing to society, for example, by taking care of grandchildren or taking care of their spouse (6, 7). To be able to support this latter positive development, health interventions focusing on promotion (8) and prevention (9) are crucial. Health promotion can strengthen a person’s capabilities and support their ability to stay healthy and master challenges in life (10). A core part of health promotion is empowerment. Empowerment is defined as gaining mastery over one’s life (11, 12), and it can be achieved with help from others or by helping oneself (13). Prevention can focus on activities that prevent risks from actually arising (9). However, a review showed that a majority of health interventions were not tailored towards older persons (14), and their prerequisites to maintain health might not be the same as for younger persons (7, 15). In order to tailor health interventions, a person‐centred perspective is needed. Person centredness has been defined as an approach where healthful relationships occur, based on persons’ values, self‐determination and mutual respect (16). There is a need for a deeper understanding of how health interventions such as a preventive home visit (PHV) can be tailored in order to be beneficial for older persons.

A comprehensive perspective including both health promotion and risk prevention is crucial in order to develop interventions which aim to maintain or improve good health (17). Risk prevention can focus on giving advice, such as having more light in order to prevent falls (18) or stopping smoking to prevent illness (19). Health promotion can focus on support through a dialogue which guides, inspires and motivates the person in regard to issues related to health (20). Many health interventions address both risk prevention and health promotion (14), and health professionals give support and advice at the same time (21). Substantial research has been conducted on risk assessments (22) and the use of assessments in different interventions in order to prevent risks such as falling (23) and malnutrition (24). However, little is known about how older persons experience the support and advice given during health interventions. Therefore, it is of importance to acquire a deeper understanding of older person’s experiences of the benefits gained from the support and advice given during interventions which aim to promote good health and prevent risks.

PHV is one health intervention with the purpose of preventing risks and promoting health among older persons (25). Previous research shows there is great diversity in how the PHVs are performed around the world (26). Usually, risk assessments are carried out using structured questions (27), and in some settings, thematic guides are used (28). As early as three decades ago, a study by Hendriksen (29) stressed the importance of having a structured conversation about risks and giving advice according to risks identified during the PHV. In addition, a review by Fagerström et al. (30) showed that the support given through a dialogue during the PHV might be important in order to also promote health. However, what is essential for older persons in such a dialogue to be able to master challenges, maintain or improve health is scarce investigated. Few qualitative studies have investigated older person’s experiences of the PHV in general (31, 32, 33). A few examples of experiences were feelings of security (31, 33), valuable help with everyday life (33) and incentives to make changes (31). However, in order to better understand how support and advice given during the PHV can be developed in order to maintain or improve good health, there is a need for research focusing specifically on support and advice. Therefore, the aim of this study was to explore older person’s experiences of the benefits gained from the support and advice given during the PHV.

## Methods

A qualitative study was conducted and analysed using content analysis (34). In order to gain a deeper understanding of the experiences of the phenomenon under exploration, interviews were chosen allowing individual perspectives to appear (35).

### Description of the context and the PHV model

This study took place in Skåne, a county in southern Sweden where seven municipalities, including both rural and urban areas, use a common model for PHVs. Visitors with different professions such as district nurses and assistant nurses conduct the PHVs. An invitation letter containing the date and time for the PHV is sent to older persons from 77 years old living at home, with no or minimal home care. The PHV then takes place in the older person’s own home and lasts for approximately two hours. The purpose of the PHV is to enable older persons to take own actions to maintain or improve health. Different aspects of health are assessed throughout the visit. The questions asked in order to assess different aspects of health cover areas such as nutrition, physical health, mental health, housing and finances. The answers are documented by the visitor in a digital support system during the PHV. The questions posed assess the older person’s health and their surrounding environment, and guide the dialogue. Depending on the answers, advice and support are individually tailored. Advice given could be about, for example, how to prevent the risk of falling, or physical exercise. Furthermore, information about services the municipality can offer and different brochures with information such as nutritional advice are provided. Support could be offered for example by talking about loneliness, and the visitor could support the person in their thoughts by listening to their story.

### Participants and recruitment

The visitors who conduct the PHVs were used as gatekeepers to get in touch with the participants. The gatekeepers were instructed to consecutively ask all who received a PHV during the time period April to May 2019 to participate in the study. The only inclusion criteria were that the participants had received a PHV. The persons received written information about the study and were invited to participate after the PHV was finished. Fifteen persons were recruited by the visitors. Due to confidentiality, we do not know how many that were asked in total. Within two weeks, each potential participant received a phone call from the first author who provided them with oral information about the study. They also had the possibility to ask questions about their participation in the study. An interview was scheduled for those who accepted to participate. Participants were allowed to choose the place for the interview, and all participants chose to be interviewed in their own homes. Two persons cancelled the interview before it had started; one did not consent to audio‐recording and the other one did not give a reason. Individual interviews with 13 older persons (Table 1) living at home were conducted by the first author.

**Table 1:** Characteristics of the participants

Characteristics	n = 13
Men/Women, n	4/9
Age, median (range)	77 (76‐91)
Cohabitant/alone, n	7/6
Highest education, n	
>3 years university studies	6
Upper secondary school	3
No upper secondary school	4
Days since PHV median (range)^a^	7 (7‐32)
Rural/urban, n	2/11

^a^ Number of days between PHV and interview. Preventive home visit (PHV).

### Data collection and procedure

Semi‐structured interviews were conducted by the first author in April to May 2019, until saturation was reached. First, two pilot interviews were conducted in order to test the interview guide, then the second and the last authors listened to the audio‐recorded interviews. Thereafter, small modifications were made to the interview guide. Before the interview started, the first author gave again oral and written information about the study and asked about informed consent. If the participants signed the informed consent form the interview started. During three of the interviews, the spouse was in the same room and in one of the interviews both participated. After ten interviews, data saturation was reached. In an attempt to confirm the saturation (36), three more interviews were made. The interviews lasted 26‐95 minutes with a mean of 53 minutes. The participants were asked to describe their thoughts about the PHV and thoughts about the support and advice given during the PHV. Probing questions were asked in order to get a deeper understanding of the participants’ experiences (35). Example of probes was ‘Can you explain more’? and ‘How do you experience that’? After the interviews, reflective notes were written by the first author. All interviews were audio‐recorded and transcribed verbatim; three interviews were transcribed by the first author and the rest by a professional transcriber.

### Data analysis

Qualitative content analysis (34) was used to analyse the interviews, and the NVivo 12 plus software was used to keep track of the codes and sort the material. All interviews were read and listened to several times by the first author. The text was divided into content areas, such as recognition, reminder, encouraging, prepared and knowledge. Meaning units with the same meaning were highlighted in the text from one of the interviews by the first and second authors separately and were then discussed and agreed upon by these authors. After that, the first author continued with the rest of the interviews in order to find meaning units connected to the aim of the study. In order to operationalise the aim of the study two research questions, ‘What do older persons experience as benefits from the support and advice given during the PHV’? and ‘How do older persons experience the benefits from the support and advice given during the PHV’? were posed to the data material. According to Graneheim and Lundman (34), every meaning unit was given a code. The coded meaning units were divided into categories answering the question ‘What’? The categories represent the manifest level (34). The first and the second authors then discussed and agreed on meaning units, codes and categories. During discussions among the authors, the theme answering the question ‘How’? emerged. The theme represents the latent level (34). Then, the last author that was not involved in the analysis validated the codes and categories. Thereafter, the interviews and the emerging findings were discussed by the first, second and last authors together. The final findings were discussed and agreed upon by all authors.

## Findings

**Figure 1:**
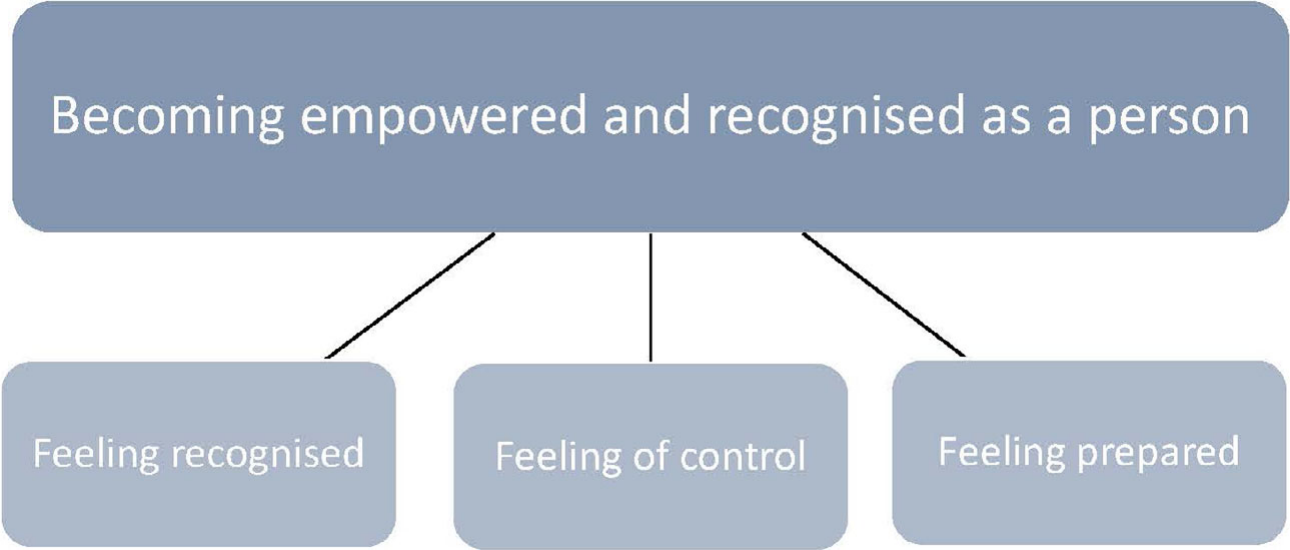
Becoming empowered and recognised as a person

The overarching theme *Becoming empowered and recognised as a person* is illustrated in the three categories *feeling recognised*, *feeling of control* and *feeling prepared* (Fig. 1). The support and advice generated conditions for the person to *become empowered* by contributing insights, reflection, reminders and information about the future. Furthermore, the support and advice contributed to a feeling of *becoming recognised as a person*, as an outcome of the support given in a dialogue and the assessment of their health, behaviour and their surrounding environment.

### Feeling recognised

The participants felt recognised by the support given during the PHV. The support was generated through them being listened to and by having the possibility to tell their story. The participants expressed a notion of older persons generally being neglected in society. However, the PHV prevented the feeling of exclusion. Instead, the support during PHV made them visible, which gave them a feeling of inclusion in society and they felt recognised as human beings. As one participant said:

Yes, I am very happy with the visit…Because it feels like someone cares, from the society, about the older…Because you feel that you are appreciated as a person, I was about to say. (Interview 3)

Furthermore, participants felt recognised and valuable when the visitor said that the person had the ability and could master challenges themselves. Participants felt that they had a lot of knowledge before the PHV, but despite this, they felt recognised for doing right. A participant who had previously worked as a nurse said:

I knew basically what information X would provide, and I also knew that it was valuable, although a lot I already knew, but it was good to get it confirmed … Well it is valuable to know that what you think is right and that in many cases you are doing the right thing. (Interview 12)

Participants expressed a feeling of being recognised for their behaviours through the support and advice generated from the assessment made during the PHV. Being assessed for one’s behaviour, such as eating habits or physical activity, led to a feeling of being recognised and resulted in a feeling of security. The participants described the support as being like a form of approval of their health and behaviour. Furthermore, some of the participants doubted their own ability to judge their health and the environment they lived in. Participants felt therefore that it was important that the visitor came and did the assessment. Their experience was that the visitor had good knowledge to assess their health, which felt important. ‘*Yes, but they can see small changes that you do not see yourself’. (Interview 10)*.

### Feeling of control

New insights emerged out of the support and advice received which generated a feeling of control. The support and advice made them reflect, and they became aware of things such as malnutrition which could be prevented with small adjustments. For example, the participants described advice such as eating late in the evening or not waiting more than eleven hours for the next meal. Advices like theses made them reflect upon their current eating habits and how they could possibly change those habits if necessary. Participants described that their new insights could lead to actions, and increase their ability to take control and manage concerns, all of which were related to health. Participants felt it was up to them to act on their new insights, take control and move to action. ‘…*I think there were many insights which were important…you have your own responsibility, you should try to take care of yourself and do as well as you can’. (Interview 5)*. Another example, one man got advice about how to modify his furniture in order to facilitate his mobility, which led to him reconstructing the furniture himself. To be aware of risks that could be prevented contributed to a feeling of control and to capability to handle different issues themselves.

Participants felt reminded of behaviours that they could change and behaviours that were good. The support and advice were experienced as a reminder, which contributed to a feeling of control, which in turn increased the participant’s ability to change unhealthy behaviours or maintain healthy behaviours. When participants were reminded of things they did well, they felt satisfied and had a feeling of believing in their own ability. Being reminded, for example of physical exercise, participants felt that they could influence their own health in a positive direction. Furthermore, participants felt reminded of how to adapt their surrounding environment, for example, in order to prevent falls. Even though they already knew it before, participants said that being reminded contributed to self‐reflection, and afterwards, for example, they decided not to get up on a chair, but instead used a ladder since it was much safer. Participants said that the support they received made them realise that they were getting older and might have to adapt behaviours in order to maintain health. The participants said that they had fallen into a pattern and things might be forgotten, so it felt good to be reminded and regain control. One woman described the support and advice as an eye‐opener, even though she had the knowledge already.

You should look out for all the practical advice, it is great that she comes with…it is a little reminder… but have you thought about this and that? I think it is a very big advantage for us old, that you get it repeated. (Interview 10)

### Feeling prepared

The support and advice contributed to a feeling of preparedness for the future in terms of expectations. Participants expressed uncertainty about the future, but the support they were given during the PHV made them feel calm. After the PHV, they knew more about what to expect of the future. The support and advice given contributed to a feeling of security when their thoughts about the future were confirmed. They felt that someone had listened to their fears, and in some cases, they got a sense of relief. An example of support which contributed to the feeling of preparedness was information about the care the municipality could provide and about what types of housing opportunities there were. One man said:

… from the information you have received you know what you can get… If for example I move into an apartment or if I come into a retirement home… I think it feels good and I like knowing that… well, you will be well taken care of…it also gives security in one way. (Interview 7)

Part of the support given during the PHV was information and contacts for future use, which made the participants feel prepared for challenges related to issues such as loneliness. If the participants encountered a problem in the future, they felt prepared with the information brochures they had received, where they could find advice about how to deal with the problem. The participants said that when life changes, for instance if losing a partner, they knew that the municipality could offer support to them. In addition, having the telephone number of a contact person working at the municipality close by, in a folder, contributed to a feeling of security. Participants revealed that they would not hesitate at all to contact this person if they wondered about something or needed help. One man said that this contact information made him feel secure: ‘*As I said, what is perhaps most valuable is that I know I can call X if there are any questions that arise’. (Interview 1)*. However, there were also participants that already knew the telephone numbers and did not feel a need for support with that information.

There was a general feeling among the participants that it was important to give the support to all older persons in order to prepare them for the future and give meaning to the future. For example, participants were aware that their situation and their lives as older persons could change fast that one day they could be in control and the next they might lose that control; therefore, it was good to be prepared. Furthermore, participants felt that the support gave them the energy and will to live, as well as faith in the future. It was positive and encouraging, and the participants felt that the visitor’s positive attitudes transferred to them. The support gave them hope for the future and participants felt boosted. One participant said that the support made her feel reassured about the future:

And just when you reach a certain age and you have a greater need and you are uncertain how the future will be, then I think it is very important that you can feel that you have someone(to contact) and that there is support to get…(Interview 3)

## Discussion

The aim of this study was to explore older person’s experiences of the benefits gained from the support and advice given during the PHV. The findings showed that participants found benefits such as becoming empowered and recognised as a person. This was demonstrated by the outcomes of the support and advice: feeling recognised; feeling of control; and feeling prepared. Through the support and advice and the subsequent new insights and increased awareness, conditions for becoming empowered were created. The participants started reflecting upon their health, which led to actions. In addition, the participants became recognised as persons through the support given in a dialogue with the visitor and as an outcome of the assessment made during the PHV.

The support and advice given during the PHVs were experienced as person‐centred. The findings from the present study revealed that older persons felt recognised as a person when the visitor listened to their stories and supported them by recognising their thoughts in a dialogue. These findings are in line with another study investigated experiences of PHVs where the persons described a feeling of being visible (31). According to our findings, participants also felt recognised as an outcome of the assessment, they described the outcome as an approval of their health. Becoming recognised is a core part of person‐centred practice (16, 38, 39). Hence, our study contributes to an improved understanding of how important it is, in health interventions with the purpose of maintaining or improving good health, to focus on recognising the person. To be able to create health interventions which are person‐centred and give advice that is tailored towards the older person’s needs, it is essential to have a supportive dialogue that gives the person an opportunity to tell their story. It allows the visitor to see the person and give advice which suits the person’s needs in order to promote good health. In line with a review by Fagerström et al. (30), a supportive dialogue might be a crucial part of the PHV, partly because it promotes good health, but also because it avoids focussing only on the assessment to prevent risks. However, the present findings indicated that using the assessment as a guide for the dialogue seems to result in a beneficial experience in terms of feeling recognised as a person. It can be argued that assessments tend to set the agenda and determine what should be brought up and assessed (27) which might counteract person centredness, where the person’s needs should guide the focus. However, the participants were experiencing the outcome from the assessment as beneficial, which indicates that the assessment might be person‐centred. By giving support and advice which is person‐centred one also gets the opportunity to maintain or improve one’s own health.

Person‐centred support and advice given in a dialogue create conditions for becoming empowered. Through the support and advice, older persons felt in control when given new insights and started reflecting on what they could do in order to maintain or improve their health. A concept analysis described person‐centred empowerment as a process where the person gain knowledge, awareness and take an active part in the process (40). It has been shown in previous research that PHVs could give incentives for actions (31) and increase awareness about how to promote health (32). Our findings revealed that by being given tools during the PHV, the person expressed a feeling of being able to master challenges and maintain health. The present findings indicate that in practice the visitor can, during the PHV, create conditions for the person to become empowered. For example, by focusing the dialogue on insights, reflection and reminders about healthy behaviour seems to stimulate actions for good health. Worth notice is that persons in this study seem to be resourceful which can facilitate the process of becoming empowered. In order to empower persons who are less resourceful follow‐up visits might be required or referral to other support services. However, when it comes to empowerment, it is worth reflecting on the challenge concerning one’s ability to disempower another person. In relations, there is always some sort of embedded power (41). Examples of power relations described by Tew (41) are power over, power shared with and power transformed to another person. In order to avoid taking power over another person and the risk of disempowerment, a relationship involving shared power or giving power to another person is preferred. In order to create conditions for a person to become empowered during the PHV, a suggestion would be to transfer power to the person. In practice, it is important for the visitor to be aware of the power embedded in relationships, and the visitor should focus on creating conditions for the person to empower themselves during the PHV.

A PHV has to include both support which promotes health, and advice which prevents risks in order to create conditions for older persons to maintain or improve their health. According to our findings, older persons described both the support and advice as positive. The support gave them energy and encouraged them to master challenges. At the same time, they were aware that risks could be revealed by the assessment and they could be given advice in order to prevent such risks. However, it has been argued that prevention and promotion cannot be separated in practice (42). In the present findings, the participants were experiencing the outcome from health promotion and risk prevention as beneficial, which suggests that both perspectives have to be included in the PHV. In conclusion, to be able to promote good health and prevent risks among older persons there is a need for both support and advice which create conditions for an older person to become empowered and recognised as a person.

Turning to some methodological considerations, trustworthiness by means of credibility, transferability, dependability and confirmability (43) will be discussed. It is worth reflecting upon the sample of 13 participants. The 13 interviews were extensive, and after ten interviews had been completed, no new significant information was generated. Nevertheless, three more interviews were conducted in order to confirm the saturation. Reflecting upon the concept of duty (44), it is morally respectful to end the data collection when saturation is confirmed. Conducting more interviews could lead to unnecessary attempts to disturb the gatekeepers and the participants. The sample consisted of participants from different municipalities and these participants had a variety of characteristics in terms of gender, age, where they lived and education. This variation in characteristics among the participants was considered to increase the credibility of the study. However, the persons who participated in the study might be more resourceful and independent than the general older population. PHVs are offered to those who are living at home without home care and therefore in comparison they might be more resourceful. Furthermore, there might be persons who had negative experiences of support and advice given, aspects which are not captured in this study. The positive experiences were discussed among the authors during the data collection. To consider this, very conscious follow‐up questions were asked to capture nuances of their experiences. A description of the PHV model and a description of the context have been made in order to increase the transferability. Furthermore, the first author wrote reflective notes during the research process which could have a positive impact on the dependability. The findings are strengthened with quotations. Codes, categories and the analysis were validated separately by the authors and also jointly discussed in order to increase the confirmability. This procedure made sure that the theme and categories were valid findings.

## Conclusion

To conclude, becoming empowered and recognised as a person were experienced as central benefits from the support and advice given during the PHV. The support and advice were experienced as person‐centred, and created conditions for older persons to become empowered. The findings from the present study highlight the positive aspects of the support and advice given during the PHV, which can guide the development of health interventions and take into account the older person’s perspective. Furthermore, a combination of a supportive dialogue and an assessment of issues related to health seem to represent beneficial advice and support for older persons.

## Conflicts of interest

The authors declare that they have no conflicts of interest.

## Author contribution

The authors are justifiably credited with authorship, according to the authorship criteria. All authors have made substantial contributions to the manuscript and approved the final version.

## Ethical considerations

This study was approved by the Ethical Review Board, Lund, Sweden (reference number 2018/849), and conducted in accordance with the Declaration of Helsinki (37).

## Funding

This study is financed by external grants from Region Skåne, and the municipalities in the project ‘Preventive home visits to seniors’.
